# Gender and Age Related Effects While Watching TV Advertisements: An EEG Study

**DOI:** 10.1155/2016/3795325

**Published:** 2016-05-26

**Authors:** Giulia Cartocci, Patrizia Cherubino, Dario Rossi, Enrica Modica, Anton Giulio Maglione, Gianluca di Flumeri, Fabio Babiloni

**Affiliations:** ^1^Department of Molecular Medicine, Sapienza University of Rome, Viale Regina Elena 291, 00161 Rome, Italy; ^2^BrainSigns srl, Via Sesto Celere 7c, 00152 Rome, Italy; ^3^Department of Economics and Marketing, IULM University, Via Carlo Bo 1, 20143 Milan, Italy; ^4^Department of Anatomical, Histological, Forensic & Orthopedic Sciences, Sapienza University of Rome, Piazzale Aldo Moro 5, 00185 Rome, Italy

## Abstract

The aim of the present paper is to show how the variation of the EEG frontal cortical asymmetry is related to the general appreciation perceived during the observation of TV advertisements, in particular considering the influence of the gender and age on it. In particular, we investigated the influence of the gender on the perception of a car advertisement (Experiment 1) and the influence of the factor age on a chewing gum commercial (Experiment 2). Experiment 1 results showed statistically significant higher approach values for the men group throughout the commercial. Results from Experiment 2 showed significant lower values by older adults for the spot, containing scenes not very enjoyed by them. In both studies, there was no statistical significant difference in the scene relative to the product offering between the experimental populations, suggesting the absence in our study of a bias towards the specific product in the evaluated populations. These evidences state the importance of the creativity in advertising, in order to attract the target population.

## 1. Introduction

It is well known that the personal reaction to external stimuli is highly influenced by the emotional sensitivity of the subject [1]. In fact, despite the human capacity of conscious deliberation, many economically relevant decisions rely on automatic, fast, and effective processes, which are not under the direct volitional control [2]. Many of these processes have been shaped by evolution in order to serve social purposes [3–5] while decision-making and evaluation in economic contexts are influenced by mechanisms dedicated to social interaction. For instance, in men, the observation of different cultural objects associated with wealth and social dominance (e.g., sports cars) elicits activation in reward-related brain areas [6, 7].

In this regard, the prefrontal cortex (PFC) appears to play a pivotal role in a larger overall circuit involved in emotional and motivational processes [8]. In fact, the frontal electroencephalographic (EEG) alpha rhythm activity is frequently used to detect inter- and between-subjects asymmetries in cortical activation. Hemispheric asymmetries in the alpha frequency range over prefrontal electrodes have become widely accepted as a correlate of approach and withdrawal related motivation in basic research [9–11]. According to the “approach-withdrawal” (AW) model, analysis of the EEG power spectrum suggests a different lateralization for the anterior cerebral hemispheres in approach and withdrawal motivational tendencies and emotions [12]. Decreased alpha activity (or higher alpha desynchronization) indicates an increase in allocation of cortical activation [1, 13]. So, calculating the frontal alpha asymmetry by subtracting the right-hemispheric electrodes data minus their left-hemispheric electrodes counterparts, ln⁡(*R*) − ln⁡(*L*) [1, 14], positive frontal asymmetry values will indicate larger relative right-hemispheric power, which corresponds to larger cortical activation in the left hemisphere, and vice versa. Specifically, a relative power suppression of the alpha rhythm across the left PFC is associated with a propensity to approach a stimulus, while the relative power suppression of the alpha rhythm across the right PFC is associated with a propensity to withdraw from a stimulus. The study of the engagement/emotional impact generated by TV commercials has been investigated by EEG alpha frontal asymmetry and showed that the observation of two versions of the same advertisement produced significantly different approach reactions [15]. The same authors, Ohme and colleagues [16], studied the approach reaction as indexed by the frontal alpha asymmetry to 4 scenes belonging to three alternative commercials of the same product (a flat screen TV) each one characterized by a different kind of creative solutions. Such scenes were considered the “emotional part” of the commercials under evaluation, which had in common the same “informational part” concerning the product and the brand. Results showed that for the emotional part there was a statistical significant difference in the approach reaction towards the three commercials, but not for the informational part. In addition, difference in cerebral activity coding the pleasantness has been also observed by employing an event-related potentials (ERPs) analysis. In particular, Handy and colleagues [17] proved that visuocortical processing evoked an increase of the early positive component (called P1), at central and parietal sites, along with an increase of the later negative component (called N2), at parietal and occipital sites, related to the observation of disliked logos. Taken together, these mentioned examples bring evidences of the link between some properties of the collected EEG rhythms during the observation of TV advertisements with the overt preferences of the observers in terms of emotions.

The importance of more “objective” instruments to measure the appreciation of a product by persons other than their verbal responses is witnessed by the evidence that at least the 70% of the new products launched worldwide (including cars, shoes, and clothes), tested by traditional techniques with questionnaires or psychological interviews, fail within the first six months [18]. This happens because people do not (or are not able to) report precisely their internal perceptions when interviewed concerning product experiences or TV commercials.

Gender and age are among the most commonly used variables in marketing and consumer research. Behavioral consequences of the exposure to commercial, for instance, the food intake after watching food-related advertisements, are already object of research. Result that showed a higher vulnerability in women for eating snack food when exposed to food commercials has been reported [19]. It is worth noticing that such result is also not merely a gender difference but rather the modulation operated by the transportability within each sex [20]. However, the effect of the consumer's gender in commercials perception still needs investigation. Some evidences suggest a different perception between women and men, with a higher approach motivation in case of an advertisement of a typical feminine item (e.g., a perfume) but mainly circumscribed to particular scenes (e.g., dancing) of the video, and not providing univocal data if different videos are considered [18]. When the advertised product is an item often considered as masculine (e.g., a car), the influence of the peculiar creativity (“the plot”) of the commercial becomes evident in order to produce an efficient impact also towards women. In particular, by evaluating the emotional response indexed by heart rate (HR) and galvanic skin response (GSR) variations, results demonstrated that women produced an emotional profile presenting a major activity in the first section of the commercial that was characterized by the presence of a famous actress and three pretty little girls. This result was probably due to the identification by the women sample with the actress and by a sort of maternal reaction toward the children. On the other hand, men's emotional profile in the same observation was predominantly activated in the final section of the advertising that was related to the technical presentation of the car, probably due to the higher level of reward elicited in men by the car's engine characteristic, in comparison to women [21] (pages 101–106). The sum of these evidences suggest the importance of further investigating the gender-related commercials perception by high temporal resolution cerebral techniques, such as EEG. In addition, the use of EEG measures enables the estimation of the approach-withdrawal motivation along the development of the different scenes composing a TV commercial related to the gender issue (Experiment 1).

Besides gender, another important dimension in the evaluation of the advertising perception is related to the age of the persons. Thanks to a number of social psychology studies, it is now known that older adults struggle with avoiding potential negative outcomes as evidenced by an aversion to change [22] and nostalgia for early experience [23]. Aging research points to an association of age with making less risky decisions [24], and a measure to quantify this perspective is the concept of “loss aversion” (LA). In this concept (LA) negative stimuli have a disproportionate psychological impact relative to positive ones [25] and can be defined in general terms as the ratio of avoidance to approach measures [26]. In a recent fMRI study, no relationship has been observed between age and LA, but an increasing neural differential sensitivity of the Ventral Striatum (VS)/Nucleus Accumbens (NAc) to avoidance responses (negative valuations) relative to approach responses (positive valuations) has been evidenced with aging. These findings suggest that a central region for reward/aversion processing (VS/NAc) changes with age [27]. The same cerebral regions involved in the reward/aversion system, such as the NAc, caudate, and putamen, are also engaged in reaction to humoristic stimuli [28]. It is of interest then to investigate how the humor could be a possible discriminant factor for the appreciation of TV advertisings between different samples of relative young and older adults (Experiment 2).

The aim of the present paper is to present data to show how the variation of the EEG frontal cortical asymmetry was related to the general appreciation perceived during the observation of TV advertisements, in particular considering the influence of the gender and age on it.

Hemispheric asymmetries in the alpha frequency range (8–12 Hz) over prefrontal electrodes have become widely accepted as a correlate of approach and withdrawal related motivation in basic research [9, 10].

According to the aforementioned theoretical framework, we expect that the alpha EEG frontal asymmetry would reflect the appreciation of TV commercials, showing major (minor) activation on the left frontal lobe during the observation of pleasant (unpleasant) scenes. In other words, the activity imbalance expressed by the AW index would report higher values for pleasant/engaging scenes and lower values for the unpleasant/disengaging ones.

## 2. Material and Methods

### 2.1. Experiment  1

*Participants.* 29 healthy subjects, 13 men (35.39 ± 2) and 16 women (37.75 ± 2.5), age ranging from 25 to 54 years, have been enrolled in the study. The project involves healthy volunteers that have been paid for their performances. Informed consent was obtained from each subject after the explanation of the study, which was approved by the local institutional ethic committee. The experiment was conducted following the principles outlined in the Declaration of Helsinki of 1975, as revised in 2000.

**Figure 1:**
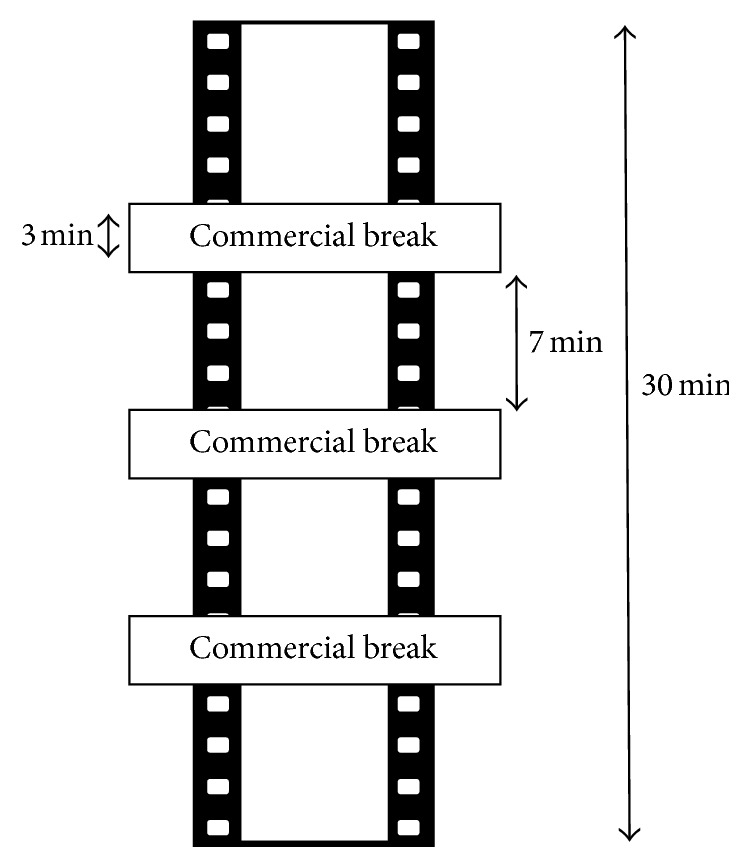
Experimental paradigm. Most of the 30-minute video consisted in nature documentary fragments, interrupted by 3 commercial breaks lasting 3 minutes each. During one of the commercial breaks (first, second, or third break, in a pseudorandomized order among participants), the target commercial was interspersed among other commercials and watched just once by subjects.

The protocol has been reported in Figure 1.

After watching the video, for each commercial three images were shown to participants, and they were asked to rate the pleasantness they would have assigned to each advertisement, ranging from 1 (total dislike) to 10 (total like).

The target commercial was a car, chosen because of the “assumed” masculine appeal of the product, but at the same time, being a family car, possibly eliciting an interest also in female observers. This feature could enable the study of the modulation by a particular creativity of the advertisement on the subjects' response in relation to their gender. The structure of the commercial, in terms of scenes, was

- 1–6 seconds: babies crying (baby cry),
- 7–11 seconds: bees buzzing (bee),
- 15–18 seconds: silence (silence),
- 21–30 seconds: music and voice of the narrator illustrating eco-incentives (product).

The target advertisement was aired by the car company Toyota and its synopsis was the comparison among auditory elements and the silent innovation developed by Toyota through its hybrid cars.

### 2.2. Experiment  2

*Participants.* 31 healthy subjects (16 men and 15 women), age ranging from 27 to 54 years, have been enrolled in the study. The participants were divided into two groups: younger adults aged <36 years (*n* = 14, mean age 30.6 ± 3.4) and older adults (*n* = 17, mean age 42.8 ± 3.1). The project involves healthy volunteers that have been paid for their performances. Informed consent was obtained from each subject after the explanation of the study, which was approved by the local institutional ethic committee. The experiment was conducted following the principles outlined in the Declaration of Helsinki of 1975, as revised in 2000.

The protocol of the exposure to the target commercial was analogue to the one of Experiment 1 (Figure 1). The specific advertisement was the one of a famous brand type of chewing gum, Air Action Vigorsol, which had as protagonists a pair of lovers living far away from each other. The specific feature of the commercial consisted in generating an exhilarating climax with some “not-so-nice” images. The structure of the target commercial, in terms of scenes, was

- 1–4 and 6–9 seconds: couple of lovers (lovers),
- 10–14 seconds: humoristic scene (air explosion),
- 5 and 15–20 seconds: appearing of the product (product).

### 2.3. EEG Recordings and Signal Processing

The cerebral activity was recorded by means of a portable EEG system (BEmicro and Galileo software, EBneuro, Italy). Informed consent was obtained from each subject after explanation of the study, which was approved by the local institutional ethics committee. All subjects were comfortably seated on a reclining chair, in an electrically shielded, dimly lit room. Electrodes were arranged according to an extension of the 10-20 international system. Since a clear role of the frontal areas has been depicted for the investigated phenomena [6, 7, 29, 30], we used the following channels: AF7, Fp2, Fpz, Fp1, AF8, F3, AF3, AFz, AF4, and F4. Recordings were initially extracerebrally referred and then converted to an average reference offline. The EEG activity has been collected at a sampling rate of 256 Hz while the impedances kept below 5 kΩ. Each EEG trace was then converted into the Brain Vision format (BrainAmp, Brain Products GmbH, Germany) in order to perform signal preprocessing such as artefacts detection, filtering, and segmentation. The EEG signals have been band-pass-filtered at 1–45 Hz and depurated of ocular artefacts by employing the independent component analysis (ICA). The EEG data have been rereferenced by computing the common average reference (CAR). Individual alpha frequency (IAF) has been calculated, according to Klimesch, for each subject, so defining the alpha band limits within the range [IAF −4, IAF +2] [31]. This calculation has been made with the purpose of relying on the personal alpha frequency, instead of the commonly adopted range 8–12 Hz, so balancing the interpersonal variability leading to inaccurate measures.

### 2.4. Approach-Withdrawal Index

In the present study, the imbalance of the EEG spectral activity in the alpha frequency band over the prefrontal areas has been chosen as the main index for the evaluation of engagement/disengagement towards the stimuli. This index was then estimated for each subject and for each condition analyzed. In particular, the approach-withdrawal (AW) index is defined as follows:

(1) $$\begin{array}{c} AW={PSD}_{R}-{PSD}_{L}. \end{array}$$

Considering that PSD is the Power Spectral Density [32, 33], which describes how the power of the acquired signal is distributed over the spectrum frequency, PSD_*R*_ represents the mean value of PSD calculated on the frontal right electrodes AF4, AF8, F4, and FP2, and PSD_*L*_ represents the average value of PSD related to frontal electrodes AF7, AF3, F3, and FP1. The AW index was calculated in each population for each movie condition.

### 2.5. Eye-Tracker Recordings and Analysis

On a subset of 12 subjects for each experiment, the eye-gaze movements have been recorded with an eye-tracker device. The eye-tracker device employed (Mirametrix technologies, Canada) returned information about the displacement of the eye-gaze for each subject during the observation of the proposed videos. Thus, the information of the collective clouds of eye-gaze in the 12 subsamples investigated is returned as “heatmaps” on the proposed videos. In particular, the computed clouds of colors will be presented on the videos from green to yellow up to red which return information on the eye-gaze density of the investigated sample. Red hotspots represent areas of the video where the majority of the eye-gazes have been concentrated. The lower the number of colored hotspots in the video, the higher the concentration of eye-gazes for the investigated sample on the same particular time instant of the video. A video with a large number of hotspots defines usually a sensory stimulation with a high number of details to be observed and then suggests a possible challenge for the sample investigated to keep the focus on the plot proposed. On the contrary, a video with few areas of eye-gaze “hotspots” suggests a very focused observation from the analyzed sample. Particular regions of interest (ROIs) were designed on the original advertising in each one of the scenes considered for this study. In these ROIs the total amount of the time spent by the eye-gaze of the subjects was estimated when compared to the total time of the duration of the scene. This information will return the attractiveness for the eye-gaze of the ROI considered. The ROIs selected for both Experiments 1 and 2 are presented in Figures 3 and 5. It could be appreciated as such ROIs considered are designed to contain the main characters of the studied advertising, such as faces or the offered product (e.g., cars and chewing gum). The percentage of the time spent for each scene by the person's eye-gaze within the selected ROIs has been taken as an index of attention of the subjects to the proposed advertising videos.

### 2.6. Statistical Analysis

For the statistical analysis of both experiments, a repeated measures Analysis of Variance (ANOVA) has been performed by using as dependent variable the neurometric AW index estimated from the different experimental setup. In particular, the average values of the neurometric indexes have been estimated along particular time interval of the video, called “scene,” as previously defined in the methods section for Experiments 1 and 2.

Different factors were considered for each of the experiments performed. In Experiment 1 the factor “GENDER” was used, with two levels (men, women) together with the factor “SCENE,” with four levels (baby cry, bee, silence, and product). In Experiment 2 the factor “AGE” with two levels (young, old) and the factor “SCENE” with three levels (lovers, air explosion, and product) were also used. For both analyses, the repeated measures ANOVA was performed with the Greenhouse-Geisser correction, to protect from the sphericity assumption violation, where the significance is set at the 5%. The post hoc tests were performed with the use of the Duncan procedure [34].

Additionally, *t*-tests have been used for pairwise comparisons between the means of the groups involved, for each of the different scenes in the two experiments.

## 3. Results and Discussion

### 3.1. Frontal Alpha Band PSD Asymmetry (AW Index)

#### 3.1.1. Experiment  1

The effect of the factor “GENDER” showed a statistical significant difference between the subgroups considered (*F*(1,27) = 4.768, *p* = 0.038), with the men group expressing higher engagement values than the women group. On the contrary, the effect of the factor “SCENE” or the interaction GENDER × SCENE did not reach the statistical significance (*F*(3,81) = 0.907, *p* = 0.441 and *F*(3,81) = 0.966, *p* = 0.415, resp.).

**Figure 2:**
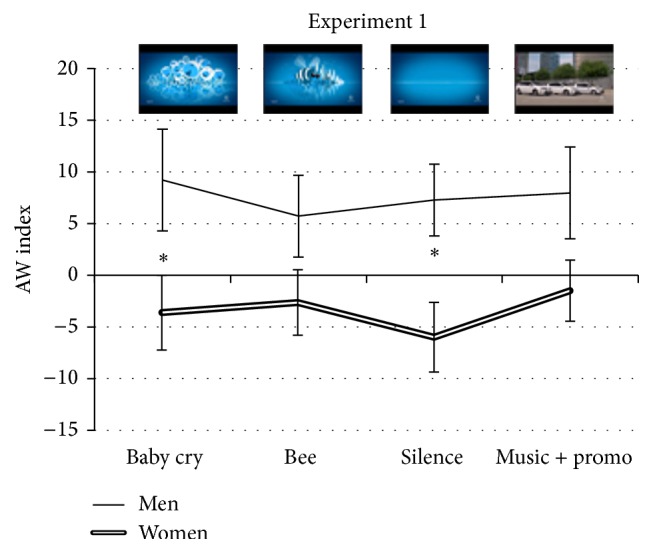
AW index expressing the PSD frontal alpha band asymmetry. The AW index showed a statistically significant difference between the groups (GENDER effect *p* = 0.038). In the comparisons between the mean of each scene reported by the two groups, a statistically significant difference was shown for the baby cry and for the silence scene. The video can be retrieved at the following address https://www.youtube.com/watch?v=7p8Oit7gbPw.  ^*∗*^Statistical significance level of *p* < 0.05.

The profile of the curves representing the average AW values reported by men and women for each scene composing the TV advertisement showed a visible different trend in the approach and withdrawal motivation between the two experimental subgroups (Figure 2). The men group expressed positive values, suggesting an approach motivation kept along all the spot development as indicated by an absence of significant difference among scenes.

The comparison between the two groups showed statistical significant higher values by men for the baby cry scene (*t*(27) = −2.056, *p* = 0.024) and for the silence scene (*t*(27) = −2.706, *p* = 0.0059). The comparison between the groups for the bee scene and for the product scene did not reach the statistical significance for few decimals (*t*(27) = −1.6, *p* = 0.06 and *t*(27) = −1.69, *p* = 0.052, resp.).

On the other hand, women showed lower and negative values for all scenes, suggesting a persisting withdrawal behavior during the observation of the entire video, also in this case deduced by the lack of significant difference in AW values among scenes.

**Figure 3:**
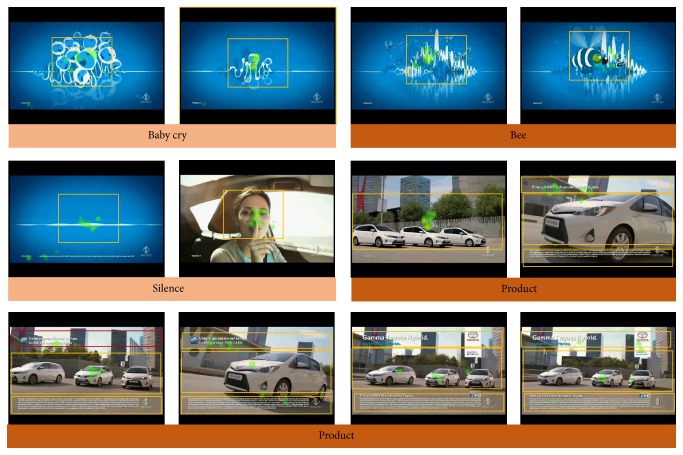
The eye-gaze observation of the investigated spot from Toyota for a subsample of 12 persons (half male, half female). Different frames of the video are presented, related to particular time segments of the advertising. The colored tape on the bottom of the images reports the name of the time segment to which the image is belonging in the Toyota video. The colored hotspots are related to the concentration of the eye-gaze of the investigated sample on the video. The orange boxes depict the regions of interest (ROIs) for the analysis. The percentage of the time spent by the eye-gaze of the investigated sample in each one of the ROIs has been analyzed.

The information from the eye-tracker measurements for the particular spot has been presented in Figure 3. We noted that such information comes from a subset of the total sample analyzed for this spot (e.g., 12 subjects). From Figure 3 it can be observed that the distribution of the eye-gaze across the different segments of the videos is all quite concentrated in the first segments of the spot. The boxes in the images describe the ROIs considered for the analysis. In particular, the values of the percentage of the time spent in the ROIs across the different segments have been presented in Figure 6(a) for the advertising observed. The first two segments of the video considered (namely, the “baby cry” and “bee”) are characterized by a low number of hotspots and by a high percentage of the time spent in the ROI analyzed. This suggests a high focused observation of the video by the investigated sample. On the contrary, during the time segments related to the product presentation (namely, “product”) the number of hotspots increases, due to the presence of the written text and multiple occurrences of cars, and this is reflected by the low amount of the index considered for such ROIs.

#### 3.1.2. Experiment  2

The effects of the between and within factors did not show statistical significant differences for the AW values between the younger adult and older adult groups. In particular, the factor AGE (*F*(1,29) = 1.458, *p* = 0.237) as well as the factor SCENE (*F*(3,81) = 2.135, *p* = 0.078) was not significant. The interaction AGE × SCENE (*F*(3,81) = 2.156, *p* = 0.10) was also not significant. On the other hand, the comparison between groups for the mean of the different scenes showed statistically significant lower engagement values for the older group (*t*(29) = −2.093, *p* = 0.046) in the air explosion scene.

**Figure 4:**
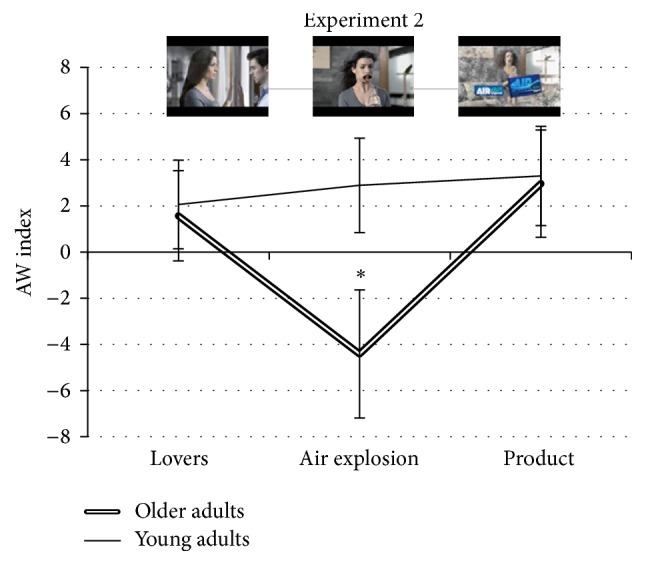
AW index expressing the PSD frontal alpha band asymmetry. The AW index did not show any statistically significant difference between the groups, while in the comparison between the means of each scene a statistically significant difference has been reported for the air explosion scene. The video can be retrieved at the following address https://www.youtube.com/watch?v=o8RCOb3WQOs.  ^*∗*^Statistical significance level of *p* < 0.05.

The pattern of the AW values expressed by the younger adult group is represented in Figure 4. Such figure shows the persistence of an approach tendency indexed by positive values during the whole succession of the scenes and presenting no statistical significant differences among the scenes. Instead, the older adult group reported a reduction of the approach motivation level from positive AW values towards negative ones in the second scene, characterized by the humoristic situation. In the same group, AW values increased in the transition to the third scene, and even if without reaching the statistical significance, the profile of the AW values for the three scenes in general suggested a trend of strong decrease for the second one in the older adults.

**Figure 5:**
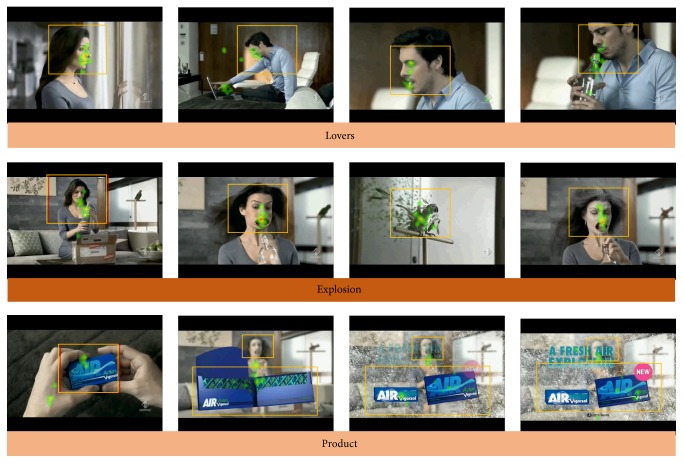
The distribution of the eye-gaze in the analyzed sample for the spot of Vigorsol considered in Experiment 2. The same convention for the representation of hotspots of Figure 3.

The information returned from the eye-tracker measurements on the particular spot investigated in this experiment is presented in Figure 5.

**Figure 6:**
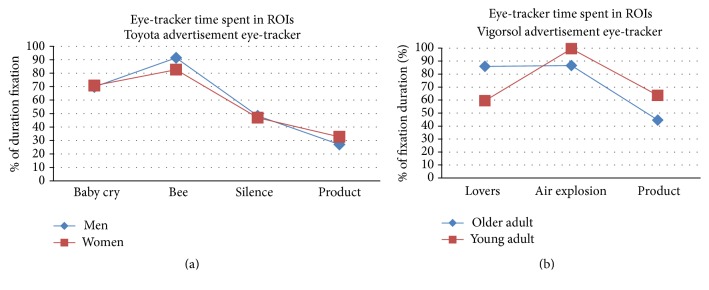
Time spent by the eye-gaze from the sampled population in the ROIs described in Figure 3  (a) and Figure 5  (b). Values expressed as percentage of the total time duration of each scene. The higher the percentage, the higher the attention of the persons within the ROI considered. (a) shows the values of the eye-gaze percentages for the Toyota advertising, as measured for each scene considered. (b) shows values of the eye-gaze percentages for the Vigorsol advertising.

In such figure, it can be noted that the distribution of the eye-gaze across the different segments of the videos is remarkably concentrated.  Figure 6(b) returns the information about the time spent on ROIs for the analyzed advertising by the sampled population. In particular, it could be noted that all these percentages are well above the chance level, as a sign of a focused attention across all the video.

In particular, the first two segments of the video considered (namely, the “lovers” and “explosion”) are characterized by the presence of one hotspot related to the male or female character that appears on turning on the screen (together with the parrot also). This suggests a high focused observation of the video by the investigated sample. Only during the time segment related to the product presentation (namely, “product,” in Figure 5) the number of focused hotspots slightly increases, due to the presence of the written text.

### 3.2. Questionnaire Results

#### 3.2.1. Experiment  1

No significant difference was shown by the comparison between the means of the pleasantness rating provided by the men (mean 6.2 ± s.d. 0.4) and by the women group (mean 5.9 ± s.d. 1.2), *t*(27) = −0.27, *p* = 0.918.

#### 3.2.2. Experiment  2

There was a statistically significant difference between the ratings provided by the younger adults (mean 8.2 ± s.d. 0.7) and by the older adults group (mean 3.9 ± s.d. 2.2), characterized by a lower value reported by the second one (*t*(29) = 6.1, *p* < 0.0001).

## 4. Discussion

### 4.1. Experiment  1

The different perception reported by men and women in Experiment  1 suggested (1) a higher engagement in men for the present product and (2) a different reaction to auditory (sounds and silence) features of the commercial. A difference in advertisements perception between men and women is witnessed in literature also by behavioral data. For instance, examining the rating of men's, women's, girls', and boys' magazines, the most preferred advertisement for women was pleasant and active, while for men unpleasant and active [35]. Furthermore, considering a very specific example as the influence of gender on the impact of food advertising, results are not univocal and need further investigation. In particular, studies found an increase in women food consumption and in men food intake, but also other researchers did not find any difference between men and women in the food intake while watching food advertising (for a review [36]). Concerning the first point, probably the presence of the written “toyota.it” on the bottom left side of the screen during all the advertisement biased the engagement reflected by the AW index towards a “male” interest [6, 7]. This could explain the difference between the groups already at the very beginning of the commercial. The counterpart of this evidence is represented by the higher AW highlighted by women during the whole length of a female perfume commercial, a product typically addressed to women [18]. The lack of a correspondence between the electroencephalographic data and the behavioral data, not expressing a difference between men and women answers, can be explained by the evidence that not all the physiological processes are under the volitional control [2], so reaching the consciousness. Additionally, the same researchers found that after an analysis of different scene, the AW values for the intro were higher for men than for women; this could explain the significantly higher value reported by men for the first scene, the baby cry scene, that theoretically should rise more the women AW values than the men's ones. The cerebral response to infants in fact produces a higher activation in the mesocorticolimbic system of women in comparison to men [37]. Concerning the second point, the video had a strong auditory characterization, presenting the sound of the baby cry, then the buzzing of bees, silence, and then music. Affective states can be rapidly regulated by environmental stimuli, such as music, that indirectly modulate the brain state [38]; additionally the reaction to sounds could be descriptive of personal conditions, also from a clinical perspective [39]. The silence, defined as the absence of sound, can be used in advertising to generate attention, to elicit emotional states, and to prepare the user to the reception of the message [40]. Results of the present study suggest that this feature, the silence, in the present video could be more effective in men than in women in eliciting interest as indexed by AW value and that in women the silence feature did not manage to elicit an increase of the engagement toward the product.

### 4.2. Experiment  2

Relative to Experiment 2 the questionnaire data appeared coherent with and explicative of AW results comparisons between groups. In fact, there were statistically significant lower values rated by the older group for this TV commercial, in addition to statistically significant lower AW values for the comic scene (air explosion) in the same group. This evidence could be explained from a brain activity point of view by the engagement of reward-related regions such as the NAc, caudate, and putamen in reaction to humoristic stimuli [28].

## 5. Conclusions

The sum of the present evidences state the importance of the creativity in advertising, whose peculiar features could address the product mainly towards a particular population. Furthermore, present results are coherent with studies that highlighted the criticality of the creative/story telling part of commercials [15, 16]. The section preceding the presentation of the product has been revealed by the present results as a feasible resource to elicit the engagement in users.

However, it must be noted that there is a long path from the cerebral perception to the effective decision-making or to the generation of an overt preferences by the subjects. It is well known that the use of neuroelectrical information (e.g., EEG) returns only part of the active cortical neural systems in the brain, while for subcortical structures the hemodynamic or a combined approach must be preferred [41–44]. In particular, there are several cognitive layers between the cerebral appreciation of the perceived sensory inflow on the prefrontal cortices as indexed by the approach-withdrawal variables and the effective expressed verbal preferences by the subjects when asked about the same stimuli. With the cerebral measures (functional Magnetic Resonance Imaging or EEG) the activity of some of these sensory and cognitive layers could be addressed. However, the more sophisticated and cultural layers linked to the proposed perception could be evaluated through a deep investigation by taking into account explicitly the verbal component through well-constructed questionnaires related to the explicit subject's preferences. Although the existence of these overlapping layers from the perception to the decision-making could be disappointing at a first view, the information about different cognitive layers could be useful to build a more comprehensive theory of the perception in the marketing contexts [45].

From the limited perspective to improve one of the multiple aspects of the sensory perception of the commercial advertising in the selected target population, the link between verbal response and EEG activity could be used to generate a metric helping in selecting scene that could fit with the possible appreciation response of the group. This information could also be used “a posteriori” to redraw partially the advertising in order to increase the appearance of the “like” parts while depressing the “dislike” parts. Verbal questionnaire and deep interviews could then complete the general picture for improving the investigated advertising.
